# Environmental Variations in *Mycobacterium ulcerans* Transcriptome: Absence of Mycolactone Expression in Suboptimal Environments

**DOI:** 10.3390/toxins11030146

**Published:** 2019-03-04

**Authors:** Daniel Sanhueza, Jean-François Guégan, Heather Jordan, Christine Chevillon

**Affiliations:** 1 MIVEGEC, IRD, CNRS, University Montpellier, 34394 Montpellier, France; jean-francois.guegan@ird.fr (J.-F.G.); christine.chevillon@ird.fr (C.C.); 2 ASTRE, INRA, Cirad, University Montpellier, 34394 Montpellier, France; 3 Department of Biological Sciences, Mississippi State University, Starkville, MS 39762, USA; jordan@biology.msstate.edu

**Keywords:** *Mycobacterium ulcerans*, Buruli ulcer, infectious disease, ecological niche, ecological transmission, mRNA-sequencing

## Abstract

Buruli ulcer is a neglected tropical infectious disease, produced by the environmentally persistent pathogen *Mycobacterium ulcerans* (MU). Neither the ecological niche nor the exact mode of transmission of MU are completely elucidated. However, some environmental factors, such as the concentration in chitin and pH values, were reported to promote MU growth in vitro. We pursued this research using next generation sequencing (NGS) and mRNA sequencing to investigate potential changes in MU genomic expression profiles across in vitro environmental conditions known to be suitable for MU growth. Supplementing the growth culture medium in either chitin alone, calcium alone, or in both chitin and calcium significantly impacted the MU transcriptome and thus several metabolic pathways, such as, for instance, those involved in DNA synthesis or cell wall production. By contrast, some genes carried by the virulence plasmid and necessary for the production of the mycolactone toxin were expressed neither in control nor in any modified environments. We hypothesized that these genes are only expressed in stressful conditions. Our results describe important environmental determinants playing a role in the pathogenicity of MU, helping the understanding of its complex natural life cycle and encouraging further research using genomic approaches.

## 1. Introduction

*Mycobacterium ulcerans* is the etiological agent of Buruli ulcer (BU), a neglected infectious disease characterized by skin ulcers which, in the absence of early treatments, produce a wide tissue destruction, leading to disfigurement and disability [1]. BU presently affects over 30 sub-tropical and tropical countries in the Americas, Africa, and Asia [2]. In the number of human cases, BU is the third mycobacteriosis after tuberculosis and leprosy in the world showing a drastic increase in the number of reported cases in the last ten years. This is especially true in Western Africa, where the Republic of Côte d’Ivoire was the most affected country with 2533 reported cases in 2010 [3].

*M. ulcerans* (MU) has been described as an environmentally persistent mycobacterium evolved from the more generalist *Mycobacterium marinum* [4,5]. Some of the characteristics of this evolution include proliferation of insertion sequences (IS2404 and IS2604), genome reduction, pseudogene formation and the horizontal transfer of the virulence plasmid *pMUM001* that encodes the genes involved in the production of mycolactone, the lipid toxin responsible, in part, for BU ulcers in humans. These genomic modifications are probably an evidence that MU is adapting to a new ecological niche [6,7,8,9,10].

Genomic analyses about the metabolic pathways affected during its reductive evolution led inference to the type of natural environment where MU could be more adapted. Indeed, the loss of the ability to express the pigments that protect *M. marinum* from sunlight damage and the lack of functional anaerobic respiration pathways suggest that MU is more adapted to a dark and aerobic or microaerophilic habitat [6,7]. Isolating live MU cells directly from the environment is unfortunately a difficult task because of the very high probability of contamination by many co-existing micro-organisms that grow faster than MU [11]. As a result, the presence of MU is based on PCR-amplification [8,12,13].

Recent data have pointed out the positive impact of human-disturbances (e.g., deforestation, dam construction, rice cultivation, etc.) onto the settling of MU in aquatic ecosystems (e.g., for comprehensive reviews [2,3,14]). In addition to this close association between aquatic environments and MU, in vitro experiments have recently reported the pivotal role of a few biotic or abiotic factors [15,16,17,18], although the environmental determinants on MU growth and persistence are not fully understood.

We are presently proposing to glean more information using a fully different approach—next generation sequencing (NGS) and mRNA sequencing [19] to analyze the changes in the MU transcriptome across conditions known to be suitable for MU growth. According to Reference [16], we choose the concentrations of either starch, calcium, chitin, or of both calcium and chitin that were previously demonstrated to correspond to the best conditions for MU growth in vitro. We did so in order to investigate the differences in MU transcriptome induction by each of these modifications of the standard growth medium (i.e., the control). It is noteworthy that previous investigations of the differences in MU growth among these modified and control conditions revealed two groups: Supplementing the culture medium in either chitin alone or in both chitin and calcium defined the most suitable conditions for MU growth, while all other conditions defined a sub-optimal group regarding the exponential phase in MU growth [16]. As the MU genome remains poorly annotated, we anticipated a difficult task to translate the observed differences in genetic expression profiles into differences in cellular functions and/or metabolic pathways. However, MU has a phylogenetic relative [20] whose genome has achieved much more scientific interest: *M. tuberculosis*. We took this advantage to run a second analysis as follows—the MU differentially expressed genes across in vitro environments were blasted against the *M. tuberculosis* (MT) genome and it is then in MT physiological pathways that we searched for the putative identity of cellular functions and/or in the metabolic pathways impacted by the MU changes in expression profiles.

## 2. Results

At 33-days post inoculation, bacterial loads were estimated by Real Time PCR, as described in Reference [16], as 1.99 × 10^5^ ± 3.38 × 10^5^ cells/mL in control, as 5.01 × 10^5^ ± 7.06 × 10^5^ cells/mL in the starch-supplemented condition, as 1.17 × 10^6^ ± 1.52 × 10^6^ cells/mL in the calcium-supplemented condition, as 1.48 × 10^6^ ± 1.76 × 10^6^ cells/mL in the chitin-supplemented condition and as 7.53 × 10^6^ ± 9.62 × 10^6^ cells/mL in the condition supplemented in both calcium and chitin (Figure S1). Overall, the MU strain 1G897 expressed 5074 genes, with 81 (1.6%) present on the *pMUM001* plasmid and 4993 (98.4%) on the bacterial chromosome.

### 2.1. Differences in Gene Expression in Experimental Environments Relatively to Control Condition

**Figure 1:**
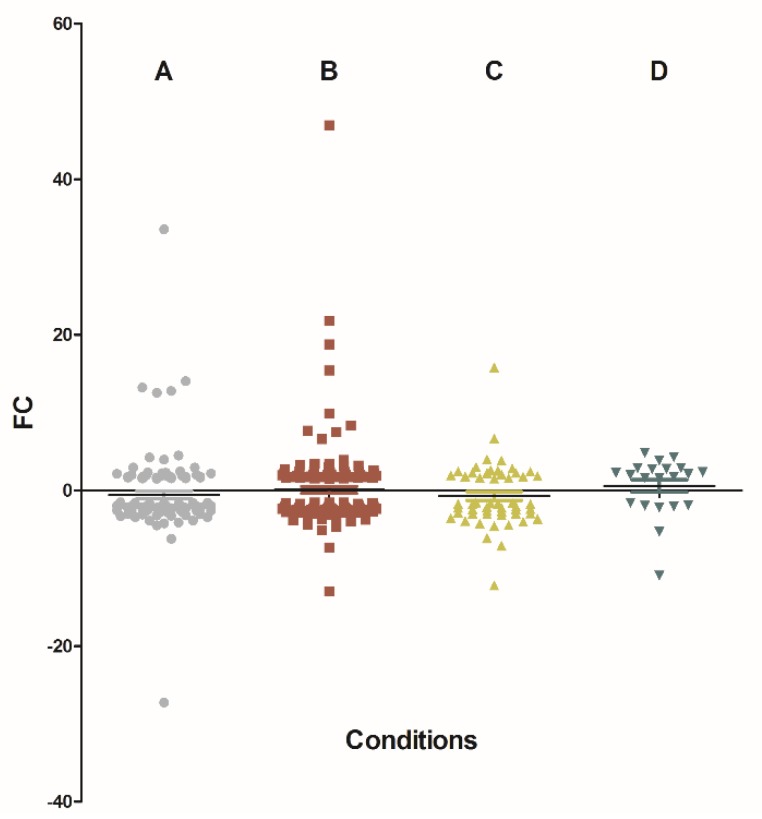
Differentially expressed genes in each of the four conditions compared to control. (**A**) is Calcium vs. control condition; (**B**) is Chitin vs. control condition; (**C**) is Chitin + calcium vs. control condition; and (**D**) is Starch vs. control condition. Each point represents a gene. On the Y axis, values above and under zero represents overexpression and under-expression, respectively.

**Figure 2:**
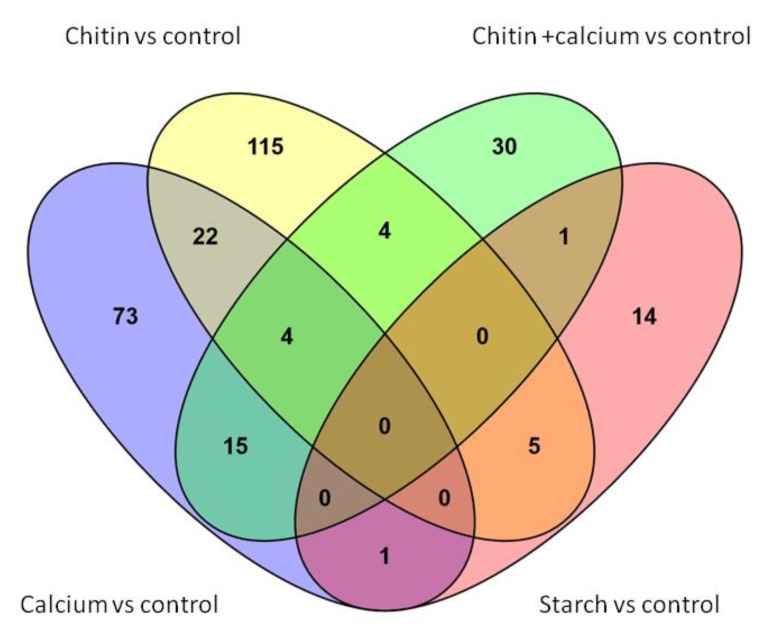
Diagram showing crossed results of each comparison performed between the four conditions used in our study and the control medium 7H9.

Overall, 340 genes were significantly differentially expressed in at least one comparison between a modified environment and the control. Relatively to control, the genes were mostly down-regulated in the conditions supplemented in either calcium alone, chitin alone or in both chitin and calcium (69.5, 64.7 and 63.3%, respectively) but up-regulated at 66.7% in the starch-supplemented environment (Figure 1). Also, the numbers of significantly under- or over-expressed genes were 115 in the calcium-supplemented environment, 150 in the chitin-supplemented environment, 54 in the environment supplemented in both chitin and calcium and 21 in the starch-supplemented environment (Figure 2). Table S1 details for each comparison and all the information regarding the identity of the genes, the amplitudes of the changes in expression, the associated *p*-values, and the knowledge available on the expressed proteins.

### 2.2. Plasmid Genes Involved in Mycolactone Production

The metabolic pathway leading to mycolactone synthesis involves six genes carried by the *pMUM001* plasmid: *mls*A1, *mls*A2, *mls*B, *MUP*053, *MUP*045, and *MUP*038. Three of these genes (*MUP*053, *MUP*045 and *MUP*038) were expressed without any change in expression level between control and any modified conditions. By contrast, no RNA corresponding to the genes *mls*A1, *mls*A2 and *mls*B was recovered in control, as in any modified environment.

### 2.3. Utilization of Chitin by M. ulcerans and Chitinases Expression

MU possesses three chitinase enzymes (genes: *MUL*_2681, *MUL*_2210, and *MUL*_0371), i.e., hydrolytic enzymes able to break down the glycosidic bonds in chitin. *MUL*_2681 was expressed at the same level in all experimental conditions surveyed. Both *MUL*_2210 and *MUL*_0371 were significantly down-regulated in chitin-supplemented growth medium relatively to the control (fold changes estimated as −1.51 and −2.46, respectively), but remained expressed at the same level as the control in all other comparisons (including the comparison between the control and the growth-medium supplemented in both chitin and calcium).

### 2.4. Biological Components Impacted by the Changes in Gene Expression

The comparison between control and the starch-supplemented growth medium did not identify any metabolic pathway affected by the observed changes in expression profiles (Table 1). For the three other comparisons, the significant detected changes more often affected a biological pathway than a molecular function (χ^2^ = 11.47; *p*-value = 0.022).

Different metabolic pathways were statistically enriched in the calcium-supplemented medium relatively to the control: namely, the macromolecular complex subunit organization (*p*-value = 0.0039), the cellular macromolecular complex subunit organization (*p*-value = 0.0074), translation (*p*-value = 0.029), and marginally, the nucleotide biosynthetic process (*p*-value = 0.054). Meanwhile, two molecular functions were enriched in the calcium-supplemented medium relative to the control: The *N*-acetyltransferase activity (*p*-value = 0.0038) and the *N*-acyltransferase activity (*p*-value = 0.0048) (Table 2A).

Relative to control, the chitin-supplemented environment significantly affected the process of DNA replication (*p*-value = 0.0068) through the modulation of four different genes (Table 2B). This comparison also showed that the chitin-supplemented growth medium significantly affected genes involved in two molecular functions; namely, carbonate dehydratase activity (*p*-value = 0.033) and zinc ion binding (*p*-value = 0.044) (Table 2B).

Finally, two metabolic pathways were enriched in the chitin-and-calcium supplemented growth medium relatively to the control: the nucleoside metabolic process (*p*-value = 0.0135) and the cellular metabolic compound salvage (*p*-value = 0.0302) (Table 2C).

### 2.5. Additional Information Driven from MU/MT Comparisons

Irrespective of the threshold applied, no significant result emerged when comparing the environments supplemented in either calcium or starch to control (Table S2).

A threshold of 50% from within the chitin-supplemented condition allowed detection of two up-regulated genes involved in the application of the biological pathway of 2-Methylnaphthalene degradation (*p*-value = 0.038), two up-regulated genes involved in the pathway described as ‘Universal stress protein A’ (IPR006015) (*p*-value = 0.039), and two up-regulated genes associated to the biological pathway of universal stress protein (IPR006016) (*p*-value = 0.040). Increasing the threshold to 75% reduced the list to the 2-Methylnaphthalene degradation that remained significantly (*p*-value = 0.0383) up-regulated relative to the control. No statistical signals remained when applying a 90% threshold (Table S2).

Applying a 50% similarity as a threshold allowed identifying in the environment supplemented in both chitin and calcium, four genes involved in the biological process of transmembrane (*p*-value = 0.027) that were differentially expressed among conditions (two downregulated and two upregulated in the supplemented medium relatively to control). Interestingly, all four genes were associated with the biological process of trans-membrane. No biological processes remained statistically significant for higher similarity thresholds than 50% (Table S2).

## 3. Discussion

Our study explored how the transcriptome of MU cells varied across culture growth media using both NGS and mRNA sequencing. This approach allowed overcoming the acute lack of awareness and understanding about gene function for this mycobacterium: The MU genome involves 4473 coding DNA sequences among which only 3229 are annotated and 1244 (27.8%) remaining genes coding for hypothetical proteins. The high variations in the estimates of MU cells across replicates presently observed at date t = 33 days post-inoculation are not surprising; similar results were observed at individual dates even if MU growth kinetics significantly differed among culture conditions when taking several dates into consideration [16]. Due to the scarcity of information and with the specific purpose to obtain more information about the MU transcriptome, we benefited from the opportunity of a comparison between the proteins produced by MU with those described in its close relative *Mycobacterium tuberculosis* (MT) (for full details see Materials and Methods). By comparison, the genome of MT involves 4158 coding DNA sequence (CDS), among which only 513 (12.3%) are not yet annotated. This allowed identifying the potential function of 10 deregulated genes in the chitin-supplemented conditions (i.e., either only in chitin or in both chitin and calcium) relative to control, and that were associated to cellular stress and the trans-membrane processes (Table S2). Thus, due to the lack of knowledge on MU genes function, these associations were derived from MT/MU comparison. None of these comparisons remain significant when evaluated by using the conservative Bonferroni’s correction procedure (Table 2). Further research describing new transcriptomic data, gene function and functional assays of MU seems indispensable to both clarify and confirm present results and improve several aspects of the existing scarce knowledge on MU.

These data indicate that MU phenotypes result from a complex combination of factors, including gene content, environmental conditions, and differentially transcriptional regulation. Obviously, the transcriptome of any bacterium depends on the environment where it has grown. Thus due to the differences in the growth medium used, it is not surprising that our results differ from another article studying the MU gene expression profiles [15]. In fact, none of the 10 significantly differentially expressed genes across the four present comparisons between modified and control culture conditions (Table 2) were differentially expressed or modulated in Deshayes et al. experiments [15]. These authors found an association between the production of mycolactone and that of mycobactin siderophores i.e., small compounds whose primary function is to chelate the ferric iron Fe^3+^ from external habitats, thereby making it available to the microbes [15]. This appears not to be the case in our experiments where the three genes *MUL*_3902, *MUL*_1209, and *MUL*_1210 (iron regulated transporters or associated to a secretion system required for surveying cellular proteins during infection) are well expressed, but not modulated between control and treatment groups. One conclusion by Deshayes et al. [15] was that the production of mycolactone by MU cells may depend on the availability of nutrients in the environment, and they indeed observed a variation in the production of mycolactone in the presence of different types of carbohydrate molecules. Carbohydrates can be present in nature in complex forms such as polysaccharides including starch, cellulose, or chitin, but also as mono and di-saccharides such as glucose or sucrose. Efficient use of available different carbohydrates by MU cells may be determined by the different types of carbohydrate transporters present in the bacterium genome and its plasmid. In addition, the expression and regulation of sugar utilization systems can also be affected by environmental conditions such as oxygen availability, temperature, or carbohydrate availability. This hypothesis remains to be directly tested: In Deshayes et al. [15] experiments, there was no carbohydrate, but starch can be considered as direct nutrients for MU, and our experimental setting was also not designed to test the effect of different sugar nutrients on MU growth and MU resource optimization.

No pattern clearly emerged when comparing across modified conditions the biological processes that were impacted in each of them. Clearly, additional knowledge on MU genomic/phenotypic relationships required to fully capture the connections between the combined and separate actions of calcium and chitin onto MU transcriptomic. Nevertheless, our results represent a good start for understanding these processes and physiological responses. Indeed, the search for the biological processes impacted in these supplemented conditions showed an association with the genes involved in the production of DNA (Table 2), probably because we made our experiments during the phase of exponential growth. We also found an association with several genes involved with the production of elements of the bacterial cell wall (in Table 2). This seems entirely logical because, in general, *Mycobacterium* species spend a lot of energy producing cell membranes, which are known for their unique architecture conferring high biocide and antibiotic resistance properties, while hydrophobicity facilitates nutrient acquisition, biofilm formation, and spread by aerosols [21].

In the present work, one of our major goals was to test whether some specific environmental conditions could promote more virulent MU phenotypes, via an over-production of mycolactone, measured in our study by the over-expression of the genes carried by the plasmid *pMUM001*. This hypothesis was presumably rejected here since mycolactone was not expressed by MU cells in any in vitro conditions tested in our experimentations. However, quantitation of mycolactone production will be necessary to confirm this. The specific function of mycolactone in nature remains unclear. Mycolactone is known to be a major virulence factor causing the human skin ulcers characteristics of BU disease [22,23], as key in the colonization of the salivary glands of water bugs (family Naucoridae) in laboratory conditions [24] and as a potential fungal chemoattractant [25]. It seems plausible that the expression of the virulence genes might be associated with stress conditions, as the colonization of new habitats such as the subdermal layer of human skin or salivary glands in water bugs. If so, it is plausible that our in vitro conditions were not propitious to stimulate the expression of the genes involved in the mycolactone metabolic pathway. Perhaps, a more stressful habitat for MU growth than those presently tested would have been more accurate for testing the possibility of variations in the production of mycolactone across environmental variations. One plausible explanation could be the existence of a negative trade-off between cell growth and mycolactone production, so that the best conditions for MU growth would correspond to the worse conditions for mycolactone production. This has been observed in laboratory cultures of MU alongside mycolactone isolation, where higher cell concentrations have been associated with a lower production of mycolactone (H. Jordan, personal communication). Furthermore, the effect of stressful environmental conditions and toxins production was established for bacteria achieving dormancy through sporulation such as *Bacillus sphaericus, B. cereus,* or *B. thuringiensis* [26,27,28]. These species produce spores embedding crystallized toxic proteins only in environments where the availability in nutrients becomes highly restraint. For *B. thuringiensis* for instance, the ingestion of dormant spores by insects leads to the insect death and thus to a new source of nutrients suitable for the bacterial germination and cell growth [26]. Accordingly, sporulation and cell growth must be balanced to optimize the bacterium survival in variable environments i.e., to ensure that the co-occurrence of both life-history strategies across unpredictable fluctuations in the availability of nutrients. In *B. subtilis*, the spore yield and viability result in a quantity versus quality tradeoff that drives the emergence of complex phenotypic traits [28]. Further research on MU should include this line of research and thus investigate the multiple tradeoffs that may exist for MU cells to survive and adapt to heterogeneous habitat conditions. It is also interesting that our experimental study indicates that MU cells can switch off the metabolic pathway leading to genes encoding for the production of the mycolactone toxin, while maintaining the carriage of the plasmid, at least in the 1G897 strain, since the joint losses in plasmid and pathogenicity were reported in another MU strain [29]. As a direct consequence, the PCR characterization of the presence of the plasmid does not necessarily mean the presence of toxic MU cells since the genetic expression of mycolactone seems to be partly under environmental-control.

Concerning the understanding of the chitin usage by MU, our results appear somehow counterintuitive since the chitinase enzymes are comparatively less expressed in chitin-supplemented conditions. From then, the first explicative hypothesis for the chitin-effect on MU growth was that MU could be able to degrade chitin and use some deriving products as nutrients [16]. Under this hypothesis, we expected to observe a higher expression of chitinase enzymes in chitin-enriched conditions relative to control. By reporting the opposite pattern, the present study rejected this first hypothesis. This may be because the control we chose corresponds to a suboptimal growth medium providing other nutrient sources in high enough quantities (and maybe easier to use than chitin for MU), as for instance, dextrose or glycerol. Alternatively, it is possible that MU cells do not use chitin as a basic nutrient. Further analyses are necessary to clarify the functional causes of the key importance played by chitin in the definition of the MU ecological niche. For instance, measuring the variation in the chitin concentration when MU is cultured in chitin-supplemented conditions could allow deciphering between these two explanatory hypotheses of the chitin effect on MU growth.

## 4. Conclusions

The present study evidenced the complementarity of transcriptomics approaches to MU field-surveys. Our data also suggest that the PCR-based detection of the *pMUM001* virulence plasmid does not necessarily translate into MU infection risks to humans. Indeed, the presence of *pMUM001* is a necessary, but not sufficient, factor for mycolactone to be produced. Moreover, present results indicated that, for the genes involved in the mycolactone metabolic pathway, variations in expression level should rather be searched on stressful, constrained, or sub-optimal conditions for MU growth.

It therefore seems essential to conduct more research based on NGS and confirmatory functional assays to better understand the variations in MU genomic transcription in its native environment. This avenue of research could aid in understanding the ecology and life style of this environmental mycobacterium, and the identification of the specific microhabitats favoring the presence of more virulent phenotypes of MU, hence increasing the infection risk for humans residing in close proximity to these environments.

## 5. Materials and Methods

### 5.1. Strain and Experiment Protocols

We used the MU strain 1G897, isolated decades ago from a patient from French Guiana [30], and the protocols described in [16]. In vitro cultures in control were thus performed in MGIT tubes containing 4 mL of 7H9 medium completed with 0.5 mL of MGIT Growth Supplement/MGIT PANTA antibiotic mixtures (Sigma-Aldrich, St. Louis, MO, United States). Alternative conditions corresponded to the addition to the control growth medium of either starch (10^4^ mg/L), calcium (80mg/L), chitin (5000 mg/L), or both chitin and calcium at a concentration of 5000 and 80 mg/L, respectively. We started our experiments using three replicates per in vitro condition. In all cases, experiments were performed at 30 °C with a starting inoculate of ~1000 bacteria per tube. They were stopped 33 days after inoculations in order to perform RNA sequencing at the late exponential growth phase (see Reference [16] for details).

### 5.2. RNA Extraction

Total RNA extraction was carried out using a trizol/chloroform extraction followed by a passage in Qiagen column using RNeasy Mini Kit (Qiagen, Hilden, Germany) [31].

### 5.3. RNA Sequencing

The quality control of both the RNA quantity available and its quality was based on the QuantiFluor RNA System (Promega, Madison, WI, USA) and picoRNA chip on a bioanalyser 2100 (Agilent Technologies, Santa Clara, CA, USA), respectively. After quality control, two replicates remained available for the control and the starch-supplemented growth media while three replicates remained for each of the other growth conditions. Then, bacterial mRNAs were amplified by two rounds of in vitro transcription (IVT) using the ExpressArt Bacterial mRNA amplification kit (AmpTec, Hamburg, Germany). Finally, RNA libraries were generated using the ScriptSeq Complete kit for bacteria (Illumina Inc, San Diego, CA, USA) that allows performing directional RNA sequencing. RNA-sequencing was performed by a private company (Viroscan 3D, Lyon, France). The Appendix A details the protocols that are organized into three steps: 1) quality control, 2) total RNA amplification, and 3) library generations, respectively.

### 5.4. Statistics

Comparisons in the level of gene expression were performed to compare and contrast the amount of mRNA in each of the four modified growth media relative to control growth medium. We concluded to a significant difference in gene expression in a modified environment relative to the control if and only if 1) gene coverage was 100% and the minimal sequencing depth was 1X (i.e., at least one mRNA copy retrieved per condition), 2) the mean difference between the two compared environments in mRNA amount was at least 1.5 fold, and 3) the *t*-test concluded to a *p-*value inferior or equal to the 5% risk. Given the known conservative bias of Bonferroni procedure [32], we postponed such a correction for multiple testing at the end of the analysis, i.e., after the search for the identity of the impacted biological processes (see Section 5.6 below).

### 5.5. Identification of the Cellular Component, Molecular Function and/or Processes Affected

Biological enrichment was processed using DAVID tool (Database for annotation, Visualization and Integrated Discovery) v6.7 (http://david.ncifcrf.gov). This tool calculates gene enrichment (highly associated genes with certain terms, which is statistically measured by Fisher’s test method) using number of genes belonging to a same biological pathway. DAVID gives a Gene Ontology (GO) linked to the gene enrichment, with GO potentially translating either a Cellular Component (CC), a Molecular Function (MF), or a Biological Process (BP) [33].

### 5.6. Use of M. tuberculosis Genome to Complete the Analysis

We took advantage of the *M. tuberculosis* (MT)*–M. ulcerans* (MU) close phylogenetic relationships [20] as follows. Each MU gene found to be significantly differentially expressed in one supplemented medium relatively to control was blasted against the MT genome in GenBank using the BLAST software (https://blast.ncbi.nlm.nih.gov/Blast.cgi). MU and MT genes were then translated to proteins using the EXPASYsoftware (https://www.expasy.org/) and aligned using the NEEDLE software (http://www.ebi.ac.uk/Tools/psa/emboss_needle/) in order to evaluate the similarity between the compared proteins. Three similarity thresholds (50, 75 and 90%) were arbitrarily fixed in the pairwise MU/MT protein comparisons before using the DAVID software to search for the bacterial CC, MF and/or BP potentially affected in supplemented growth media relative to the control condition.

## Figures and Tables

**Table 1:** Identification using DAVID (Database for Annotation, Visualization and Integrated Discovery) tool of the Gene Oncology (GO) affected by the environment changes and variations among treatments. Treatments refer to the comparisons between modified environments and control or to the differentially expressed genes identified when comparing to control the environments enriched in chitin, calcium or in both chitin and calcium. As DAVID do not recognize the names of *M. ulcerans* genes (e.g., *MUL*_0001), we used the Gene Identification (GI), assigned to each sequence when processed by NCBI.

Treatments	Total Number of Affected Genes	With GI N (%)		At Least One GO Retrieved
**Comparisons:**				
‘Calcium vs. Control’	73	65	(89)	YES
‘Chitin vs. Control’	115	79	(69)	YES
‘Calcium + Chitin vs. Control’	30	27	(90)	YES
‘Starch vs. Control’	14	10	(71)	NO
**Genes in common between:**				
‘Calcium vs. Control’ & ‘Chitin vs. Control’	22	21	(95)	YES
‘Chitin vs. Control’ & ‘Calcium + Chitin vs. Control’	4	3	(75)	NO
‘Calcium vs. control’ & ‘Calcium + Chitin vs. Control’	15	12	(80)	NO
All three comparisons	4	3	(75)	NO

**Table 2:** Enriched biological pathways associated to each in vitro comparison between a modified culture growth medium and the control. For each comparison, the characteristics of the gene ontology retrieved by DAVID (GO type, GO ID, GO term, associated *p-*value, fold enrichment ) are described together with the number of genes involved in each significantly affected pathway, the number of these genes that experienced a significantly change in expression in our experiments (toward up- or down-regulation in parentheses) and their name as seen in Genbank.

Treatments	GO Type	GO ID	GO Term	*p-*Value	Fold Enrichment	Genes (N)	Deregulated Genes (N)	Gene List (Expression Change)
A: Calcium vs. control	Biological Process	0043933	Macromolecular complex subunit organization	0.0039	11.481	27	4 (1 upregulated, 3 downregulated)	*MUL*_0626(2.23)//*MUL*_1353(−2.62)//*MUL*_2075(−1.56)//*MUL*_3967(−1.73)
	Biological Process	0034621	Cellular macromolecular complex subunit organization	0.0074	21.136	11	3 (1 upregulated, 2 downregulated)	*MUL*_0626(2.23)//*MUL*_2075(−1.56)//*MUL*_3967(−1.73)
	Biological Process	0006412	Translation	0.0288	3.954	98	5 (5 downregulated)	*MUL*_0073(−2.58)//*MUL*_0741(−2.74)//*MUL*_0865(−2.21)//*MUL*_2075(−1.56)//*MUL*_4181(−1.81)
	Biological Process	0009165	Nucleotide biosynthetic process	0.0537	4.366	71	4 (2 upregulated, 2 downregulated)	*MUL*_1364(2.58)//*MUL*_1784(1.83)//*MUL*_2370(−2.25)//*MUL*_3967(−1.73)
	Molecular Function	0008080	*N*-acetyltransferase activity	0.0038	11.796	23	4 (1 upregulated, 3 downregulated)	*MUL*_1771(−2.59)//*MUL*_3389(−2.51)//*MUL*_4691(1.81)//*MUL*_4933(−3.07)
	Molecular Function	0016410	*N*-acyltransferase activity	0.0048	10.852	25	4 (1 upregulated, 3 downregulated)	*MUL*_1771(−2.59)//*MUL*_3389(−2.51)//*MUL*_4691(1.81)//*MUL*_4933(−3.07)
B: Chitin vs. control	Biological Process	0006260	DNA replication	0.0068	9.472	30	4 (3 upregulated, downregulated)	*MUL*_0072(2.47)//*MUL*_0517(−3.27)//*MUL*_1222(2.61)//*MUL*_3525(2.44)
	Molecular Function	0004089	Carbonate dehydratase activity	0.0332	57.853	2	2 (1 upregulated, 1 downregulated)	*MUL*_2767 (−2.8)//*MUL*_3373(1.89)
	Molecular Function	0008270	Zinc ion binding	0.0440	3.571	81	5 (2 upregulated, 3 downregulated)	MUL_0517(-3.27)//MUL_2210(-1.51)//*MUL*_2342(1.73)//*MUL*_2767(−2.8)//*MUL*_3373(1.89)
C: Chitin + calcium vs. control	Biological Process	0009116	Nucleoside metabolic process	0.0135	15.223	24	3 (1 upregulated, 2 downregulated)	*MUL*_3335(2.22)//*MUL*_4200(−1.58)//*MUL*_4862(−3.14)
	Biological Process	0043094	Cellular metabolic compound salvage	0.0302	60.862	4	2 (2 downregulated)	*MUL*_4200(−1.58)//*MUL*_4862(−3.14)

**Figure A1:**
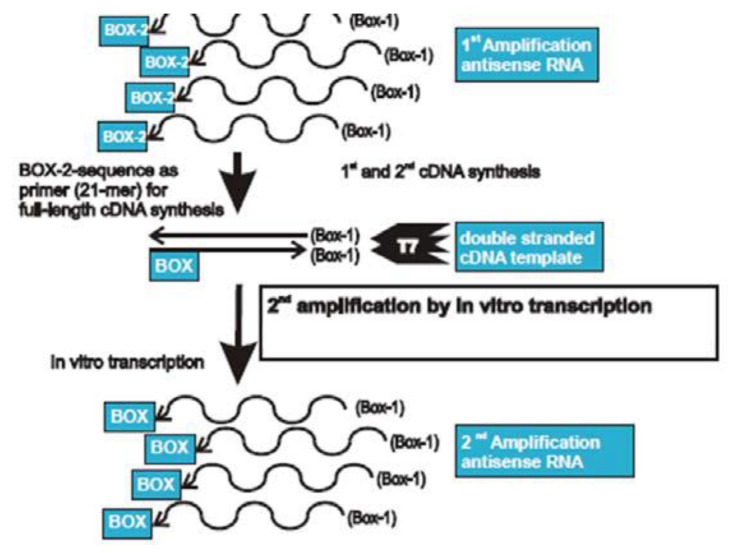
Principle of ExpressArt bacterial mRNA amplificaion system.

**Figure A2:**
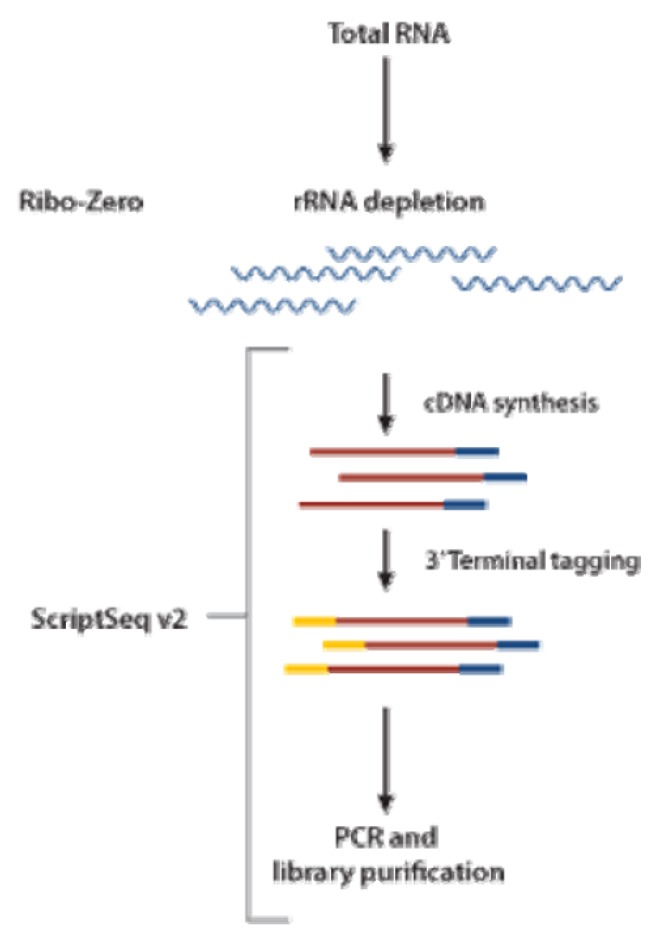
Principle of RNA library generation.

**Figure A3:**
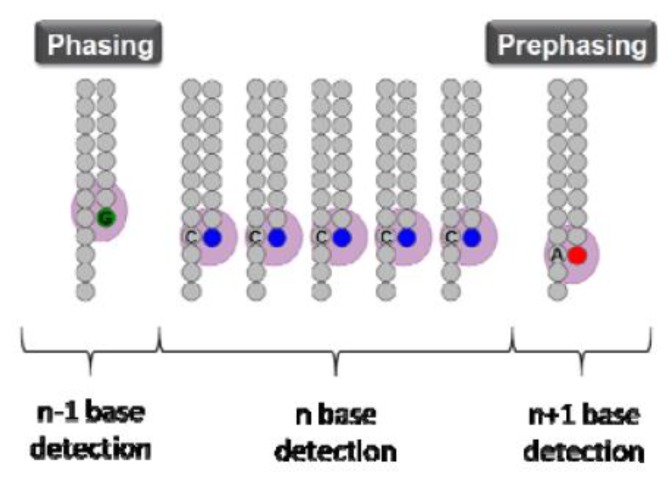
Principle of phasing/prephasing, allowing to describe how well the chemistry of the sequencing have been performed.

## Supplementary Materials

The following are available online at https://www.mdpi.com/2072-6651/11/3/146/s1, Figure S1: Box-plots illustrating the number of MU cells at day 33 in the differents conditions used in our experimental setting Table S1: Detailed information on the genes changing expression levels in modified environments relatively to control. Table S2: Putative changes in cellular component, molecular functions and biological processes driven from the knowledge acquired on *Mycobacterium tuberculosis*.

## Appendix A. Detailed RNA Sequencing Protocol

Rna sequencing technique.

### RNA Quality Control

Total RNa were quantified using QuantiFluor RNA System (Promega, Madison, WI, USA) and qualified using picoRNA chip on bioanalyser 2100 (Agilent Technologies, Santa Clara, CA, USA).

### Total RNA Amplification

Bacterial messenger RNAs were amplified by two rounds in vitro transcription (IVT) using ExpressArt Bacterial mRNA amplification kit (AmpTec, Hamburg, Germany) following supplier’s instructions.

Briefly, 1 ng of total RNA is reverse transcribed using TRInucleotide random primer. These primers are selected to not bind on ribosomal RNA, thus allowing to amplify only bacterial messenger RNA. After cDNA synthesis, the complementary strand is generated to obtain a ds-cDNA. During this step, the T7-promoter is incorporated in 3’. Then a first round of in vitro transcription is performed, thus generating antisens RNA (a-RNA). The process is repeated once, using a-RNA as a template, in order to perform the second round of IVT amplification. After this step, a-RNA are purified, quantified using QuantiFluor RNA System (Promega, Madison, WI, USA) and qualified using nano RNAchip on Bioanalyser 2100 (Agilent Technologies, Santa Clara, CA, USA).

### Library Generation

RNA libraries were generated using ScriptSeq Complete kit for bacteria (Illumina Inc, San Diego, CA, USA) that allows to perform directional RNA sequencing. Library were generated using 100 ng of previously synthesized a-RNA following supplier’s instructions with the following modifications.

Briefly, the ribosomal depletion step was skipped because of the total RNA amplification methods that excludes ribosomal RNA. aRNA were reverse transcribed without previous fragmentation because their mean size was low compare to high quality total RNA. Reverse transcription was terminated using tag in order to obtain sranded cDNA. cDNA was purified using Ampure XP Beads (Beckman Coulter, Brea, CA, USA). Then Illumina P5 and P7 sequencing adapter and a barcode specific to each sample were added during the final PCR step. Libraries were size were assessed using High sensitivity DNA chip on.

Bioanalyser 2100 quantified using QuantiFluor DNA System. Molar quantification was performed using KAPA Library Quantification Kit (Kapa Biosystems Inc, Wilmington, MA, USA) and this quantification was used to generate an equimolar pool of the fifteen libraries. Pool was loaded on two lanes of HiSeq 2500 rapid run and sequenced in paired-end of 50 nucleotides.

### ANNEX—Sequencing vocabulary

**Base call**: The base call is the process during which the sequencing machine attributes a base identity to each cluster depending on the color of this cluster. This process is performed simultaneously during sequencing for each added base.

**Cluster**: region of the flowcell containing the same sequence. Clusters are generated by a solid-phase amplification and contained about 1000 copies of the same sequence in close proximity (about one micron in diameter or less).

**Demultiplexing**: The first step of data analysis is the “demultiplexing” step. This step consists in attributing each read to the corresponding sample thanks to the index sequence. This step is performed using an Illumina Inc. Program called BCL converter. Following the demuliplexing step, attributed reads are analyzed for quality, giving the number of reads passing filters (PF reads) and the mean quality score. This step is performed using the Casava v1.8.1 from Illumina. The format of data after demultiplexing is Fastq files.

**FASTQ file**: file containing the raw sequence data (base and quality). This file is to be conserved as it contained all data about the sequencing experiment.

**Index**: specific sequence that identify sequences belonging to a specific sample. This sequence allows the “multiplexing” of sample in a single experiment.

**Phasing/Prephasing**: This quality parameter estimates the sequencing synchronization of all clones in a cluster by calculating a color-ratio.

**PhiX**: Phix is a control of sequencing process added with libraries when loaded on the lane. It comes from the Enterobacteria phage phiX174 sensu lato genome sequence.

**Qscore (Q20, Q30, and Q40):** The Qscore is a quality criteria that define the probability that a base is wrong. Three thresholds exist for Qscore: the Q20 (1 error in 100 bases), Q30 (1 error in 1000 bases) and the Q40 (1 error in 10,000 bases).

**Raw-read, read passing filter**: a read is the sequence generated by the sequencing run. Its length depend on the cycle number performed during the sequencing run. Raw-reads is the term to name a read before analysis of quality control. When a read is consistent with quality control criteria it is name a passing filter read (PF read).

**Undetermined reads at demultiplexing step**: During demultiplexing some reads are not attributed to a sample because of 1) lack of index sequence in case for example of PhiX control sequence, or 2) error in index sequence due to furnisher error in synthesis or errors during sequencing step. The undetermined reads are thus not used for further analysis [34].
