# Supplementary figures and images for: Environmental Variations in Mycobacterium ulcerans Transcriptome: Absence of Mycolactone Expression in Suboptimal Environments
Source: Toxins (Basel). 2019 Mar 4;11(3):146. doi: 10.3390/toxins11030146 (PMC6468629; doi:10.3390/toxins11030146)

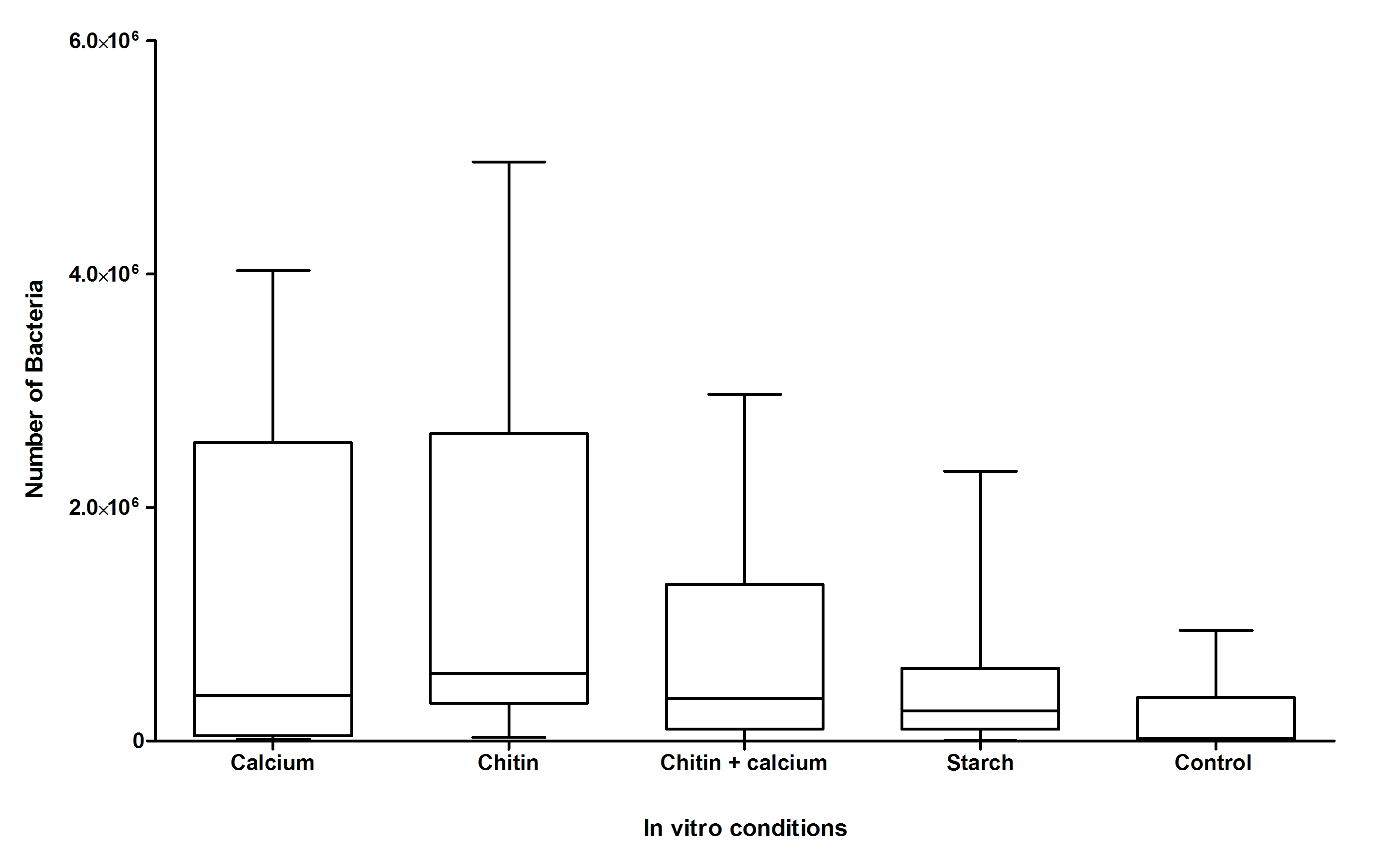

Supplement: Supplementary file 1 [file toxins-11-00146-s001.zip › toxins-446075-supple-proofreading/Figure S1.tif]
